# Probe-based confocal laser endomicroscopy for real-time evaluation of colorectal liver metastasis in resected surgical specimens

**DOI:** 10.1007/s13577-023-00965-9

**Published:** 2023-08-23

**Authors:** Mikiya Takao, Yoshikuni Kawaguchi, Masaru Matsumura, Yusuke Kazami, Meguri Tanimoto, Satoru Abe, Harufumi Maki, Takeaki Ishizawa, Junichi Arita, Nobuhisa Akamatsu, Junichi Kaneko, Norihiro Kokudo, Kiyoshi Hasegawa

**Affiliations:** 1 Hepato-Biliary-Pancreatic Surgery Division, and Artificial Organ and Transplantation Division, Department of Surgery, Graduate School of Medicine, The University of Tokyo, 7-3-1 Hongo, Bunkyo-ku, Tokyo, 113-8655 Japan; 2 National Center for Global Health and Medicine, 1-21-1 Toyama, Shinjuku-ku, Tokyo, 162-8655 Japan

**Keywords:** Probe-based confocal laser endomicroscopy, Intraoperative diagnosis, Hepatic autofluorescence, Fluorescein sodium, Colorectal liver metastasis

## Abstract

Probe-based confocal laser endomicroscopy (pCLE) enables real-time examination of tissue structure. This study investigated pCLE with or without fluorescein sodium for the intraoperative diagnosis of colorectal liver metastasis (CLM) and detection of surgical margins. Thirty-four specimens of CLM and adjacent noncancerous tissue were obtained from 21 patients and examined by pCLE between May 2017 and March 2018. Images were obtained both without and with fluorescein sodium applied to the cut surface and compared with hematoxylin and eosin-stained tissue. Fluorescence intensity (FI) was measured by luminance-analysis software. Without external fluorophores, pCLE visualized 91.2% of CLM tissues as an irregular structure with low autofluorescence and 90.5% of noncancerous liver tissues as a regular structure with high autofluorescence. The median FI was significantly lower in cancer than in benign tissue in patients without chemotherapy [70.4 (51.6–110) vs. 48.3 (39.0–59.4), *p* = 0.002] and with chemotherapy [67.9 (54.6–89.2) vs. 48.6 (28.8–82.1), *p* < 0.001]. The border was clearly visible; pCLE with fluorescein sodium clearly showed their morphologies. In summary, our study demonstrated real-time pCLE distinguished CLM and noncancerous tissue by differences in structure and FI regardless of prehepatectomy chemotherapy. Fluorescein spray facilitated clear visualization of differences in the morphology.

**Supplementary Information:**

The online version contains supplementary material available at 10.1007/s13577-023-00965-9.

## Introduction

Liver resection is the standard treatment of colorectal liver metastasis (CLM). Negative surgical margins are associated with better overall survival and lower risk of recurrence [1–7]. Intraoperative pathological evaluation of frozen sections is the gold standard for confirming surgical margins. The preparation and microscopic analysis of the approximately 6 μm thick sections requires 30–40 min and may not include all the regions that the surgeon requires. Probe-based confocal laser endomicroscopy (pCLE) has been found useful for real-time endoscopic examination for Barrett’s esophagus, and pancreatic and biliary conditions [8–12]. Our group previously reported the use of pCLE for real-time intraoperative microscopic evaluation to differentiate cancerous from noncancerous tissues in the liver by autofluorescence without need of a fluorophore [13]. It is not known whether pCLE can differentiate metastatic tissue from adjacent noncancerous liver tissue. This study investigated intraoperative pCLE to differentiate CLM from noncancerous tissue in patients with or without prehepatectomy chemotherapy and whether the use of a fluorophore improved pCLE imaging.

## Methods

This study was conducted with the approval of the Institutional Ethics Review Board of the University of Tokyo (2018001SP) and registered in the UMIN-CTR (UMIN000028667, http://www.umin.ac.jp/english/). Written informed consent was obtained from all patients in accordance with the Declaration of Helsinki.

### Patients

The subjects consisted of consecutive patients who underwent liver resection for CLM at the University of Tokyo Hospital from May 2017 to March 2018.

### pCLE

The Cellvizio 100 pCLE system (Mauna Kea Technologies, Paris, France) had a reusable imaging Demo-probe (Mauna Kea Technologies) and a laser scanning unit. The probe had the same resolution and specifications, but a different length, than those of the AlveoFlex probe (Mauna Kea Technologies). The wavelength of the blue laser light was 488 nm, the confocal depth was 0–50 µm, the field of view was 600 µm, the resolution was 3.5 µm, and the images were collected at nine frames per second. The pCLE images viewed on the liquid crystal display of a personal computer and edited with the Cellvizio software.

### pCLE and pathological evaluation of surgical specimens

Tissue specimens were immediately retrieved from the operative field after resection, cut to include the maximum diameter of the tumor and sliced parallel to the first cut surface into 10 mm sections using a long blade. The cut surfaces were evaluated by pCLE outside the operating room. The probe was manually placed perpendicular to the surface of the liver specimen, which included cancerous and adjacent noncancerous tissues. The specimen was initially examined by pCLE without using an external fluorophore (nonenhanced pCLE) to obtain images by autofluorescence [13]. The specimen was then examined after spaying fluorescein sodium (Fluorescite, Alcon Japan Ltd, Tokyo, Japan) on the cut surface and waiting 1 min before wiping it off (FS-enhanced pCLE) [14–16]. After pCLE examination, the specimens were fixed in 10% neutral buffered formalin, embedded in paraffin, sectioned, and stained with hematoxylin and eosin (HE) for pathological examination. The pathological findings and pCLE images were compared.

### Evaluation of fluorescence intensity (FI)

The FI of nonenhanced pCLE images was measured by luminance-analysis software (U11437; Hamamatsu Photonics) [17, 18]. The FI was measured in 400 µm^2^ fields within each image, and the values obtained in cancerous and noncancerous tissues in patients with or without preoperative chemotherapy were compared. FI was measured in five randomly-selected fields in each sample and reported as the mean value.

### Statistical analysis

FI values were compared between cancerous and non-cancerous regions for each patient using the Wilcoxon signed-rank test. FI ratios of non-cancerous/cancerous tissue were compared between patients with and without preoperative chemotherapy using the Mann–Whitney U test. The statistical analyses were performed using statistical software, the SPSS v 22.0 (IBM-SPSS, Chicago, IL, USA).

## Results

### Patients

Twenty-one of 42 patients with eligible surgery during the study period were enrolled in the study. The other 21 patients (50%) were excluded because pCLE system operators were unavailable at the time of surgery. The patient characteristics are shown in Table 1. The 21 patients included in the analysis had 34 lesions that were histopathologically diagnosed as CLM. Preoperative chemotherapy had been given to 13 of the 21 patients (61.9%); they provided 20 of the 34 (58.8%) lesions.

**Table 1:** Demographic characteristics of 21 patients who underwent resection of CLM

Characteristic	Value
Age, year, median (IQR), years	66 (56.75–68)
Sex, male: female, n	14: 7
Location, colon: rectum, n	12: 9
Prehepatectomy chemotherapy, n (%)	13 (61.9%)
Oxaliplatin or irinotecan, n (%)	10 (47.6%)
With anti-VEGF agent, n (%)	5 (23.8%)
With anti-EGFR agent, n (%)	5 (23.8%)
Largest liver metastasis diameter, median (IQR), cm	2.5 (1.5–3.4)
Tumor number, median (IQR)	2 (1–3)
Tumor differentiation, well/moderate: poor, n	20: 1

*IQR* interquartile range, *VEGF* vascular endothelial growth factor, *EGFR* epidermal growth factor receptor

### Structure of CLM and noncancerous liver tissue

**Fig. 1:**
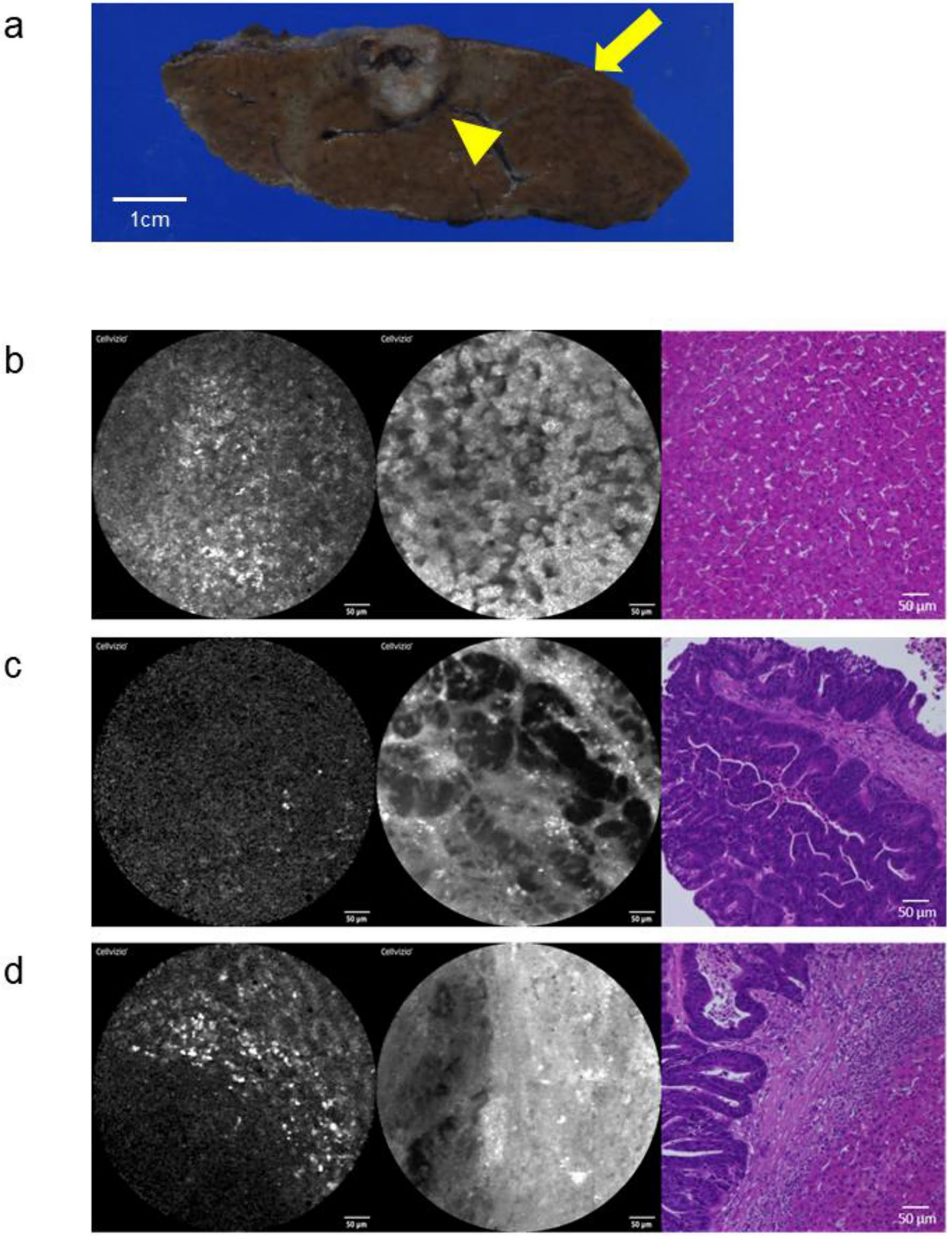
pCLE images and histopathology of hematoxylin and eosin-stained tissue in CLM patients without preoperative chemotherapy. **a** Gross appearance of a liver specimen (arrowhead, CLM; arrow, liver tissue) **b**–**d** Images without fluorescein sodium (left), after spraying of fluorescein sodium (middle), and after HE staining (right) **b** Background liver tissue with nonenhanced pCLE images showing regular tissue structure with high fluorescence (left) and an FS-enhanced pCLE image with regular arrangements of hepatocytes (middle) **c** Nonenhanced pCLE images of CLM tissue with an irregular structure and low fluorescence (left) and an FS-enhanced pCLE image showing thick tubular structures (middle) similar to the histopathology seen with HE staining (right) **d** Border of CLM and background liver tissue

The structural patterns of CLM and noncancerous liver tissue in pCLE images without and with fluorescein sodium enhancement are shown in Table 2. Representative pCLE images in CLM patients without preoperative chemotherapy (Fig. 1). A surgical specimen of partial resection of segment 7 showed a 10 mm subcapsular tumor with a round shape (Fig. 1a). Nineteen of 21 nonenhanced pCLE images of benign liver tissue (90.5%) had regular structures (Fig. 1b). Five FS-enhanced pCLE images were excluded because excess fluorescein sodium caused blown-out highlights that did not allow evaluation (Supplementary Fig. 1). FS-enhanced pCLE images of all 16 (100%) benign tissues clearly showed a regular arrangement of hepatocytes, similar to that seen in HE-stained tissue (Fig. 1b). Thirty-one of 34 nonenhanced pCLE images (91.2%) of CLM showed tissue with an irregular structure (Fig. 1c). After excluding 14 images with blown-out highlights (Supplementary Fig. 1), FS-enhanced pCLE images clearly visualized thick tubular structures in 19 of the remaining 20 CLM specimens (95.0%) that appeared similar to HE-stained CLM tissue (Fig. 1C). The borders of cancerous and noncancerous tissue were visible in pCLE images with or without external fluorophores in 32 CLM specimens (94.1%).

**Table 2:** Structures of 34 colorectal liver metastases and 21 background liver tissues in PCLE images

	Non-enhanced PCLE images		Fluorescein sodium-enhanced PCLE images	
	Structures		Structures	
Background liver tissue N = 21	Regular Irregular^a^	19 (90.5%) 2 (9.5%)	Regular Irregular (Blown out highlights^d^)	16 (100%^c^) 0 (5)
Colorecta liver metastases N = 34	Irregular Regular^b^	31 (91.2%) 3 (8.8%)	Tubular Regular (Blown out highlights^d^)	19 (95.0%^e^) 1 (5.0%^e^) (14)

*PCLE* probe-based confocal laser endomicroscopy

^a^Close to the structures of colorectal liver metastases

^b^Close to the structures of background liver tissue

^c^Of the 16 patients excluding 5 patients who showed blown out highlights

^d^Inappropriate images due to fluorescein sodium sprayed on the specimen more than necessary

^e^Of the 20 patients excluding 14 patients who showed blown out highlights

### Patients with prehepatectomy chemotherapy

**Fig. 2:**
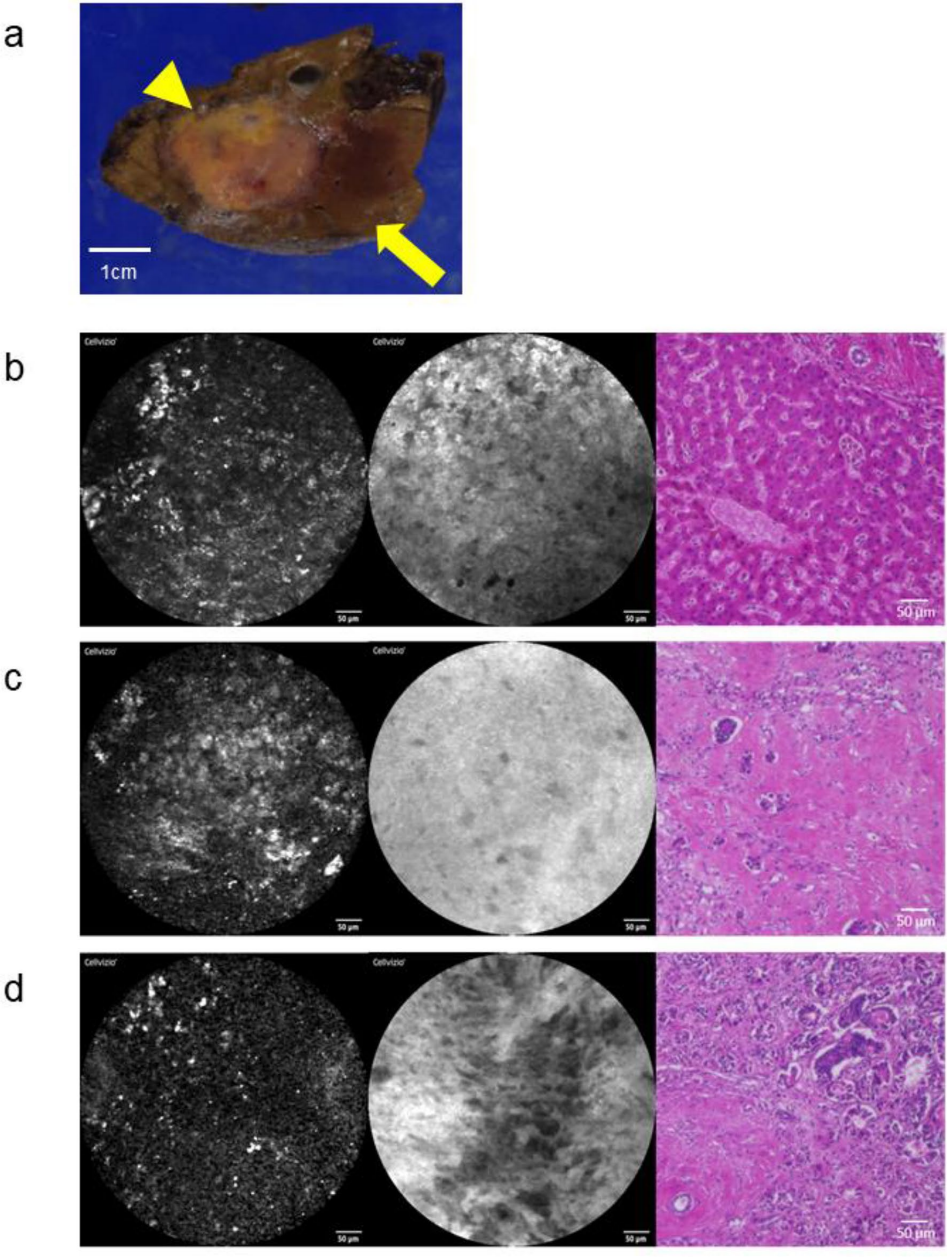
pCLE images and histopathology of hematoxylin and eosin-stained tissue in CLM patients with preoperative chemotherapy. **a** Gross appearance of a liver specimen (arrowhead, CLM; arrow, liver tissue) (b–d) Images without fluorescein sodium (left), after spraying fluorescein sodium (middle), and after HE staining (right) **b** Background liver tissue with pCLE images of background liver tissue similar to those of patients without preoperative chemotherapy: without fluorescein sodium (left) and with fluorescein sodium (middle) **d** In the center of a CLM free of malignant cells after chemotherapy, nonenhanced pCLE shows relatively high fluorescence (left); FS-enhanced pCLE with structures (middle) that corresponding to the fibrotic changes seen with HE staining (right) **d** In the periphery of a CLM including residual malignant tissues after chemotherapy, background liver tissue and CLM were differentiated without or with fluorescein sodium, as in patients without chemotherapy

Representative pCLE images obtained in tissue from the 13 patients (61.9%) with four courses of mFOLFOX6 plus an anti-EGFR agent before surgery are shown in Fig. 2. Surgical specimens of partial resection of segments 7 and 8 showed a 31 mm diameter tumor with an irregular shape and fibrotic changes (Fig. 2a). pCLE images of the surrounding liver tissue (Fig. 2b), the center of the CLM (Fig. 2c), and the periphery of the CLM (Fig. 2d) were similar to those shown in Fig. 1. Nonenhanced pCLE images of noncancerous liver tissue included regular structures with high fluorescence (FI = 62.7, Fig. 2b, left). HE staining showed prominent sinusoidal dilatation in response to oxaliplatin-based chemotherapy (Fig. 2b, right). Nonenhanced pCLE images of the center of a CLM showed irregular structures with high fluorescence (FI = 72.4, Fig. 2c, left). FS-enhanced pCLE images showed neither tubular structures nor regular hepatocyte-like structures (Fig. 2c, middle). HE-stained tissue showed fibrotic changes with a few cancer cells (Fig. 2c, right). Nonenhanced pCLE images of peripheral CLM tissue showed irregular structures with low fluorescence (FI = 41.8, Fig. 2d, left). FS-enhanced pCLE images showed deformed tubular structures (Fig. 2d, middle) that corresponded to tubular structures visible in an HE image (Fig. 2d, right).

### Fluorescence of CLM and surrounding liver tissues

**Fig. 3:**
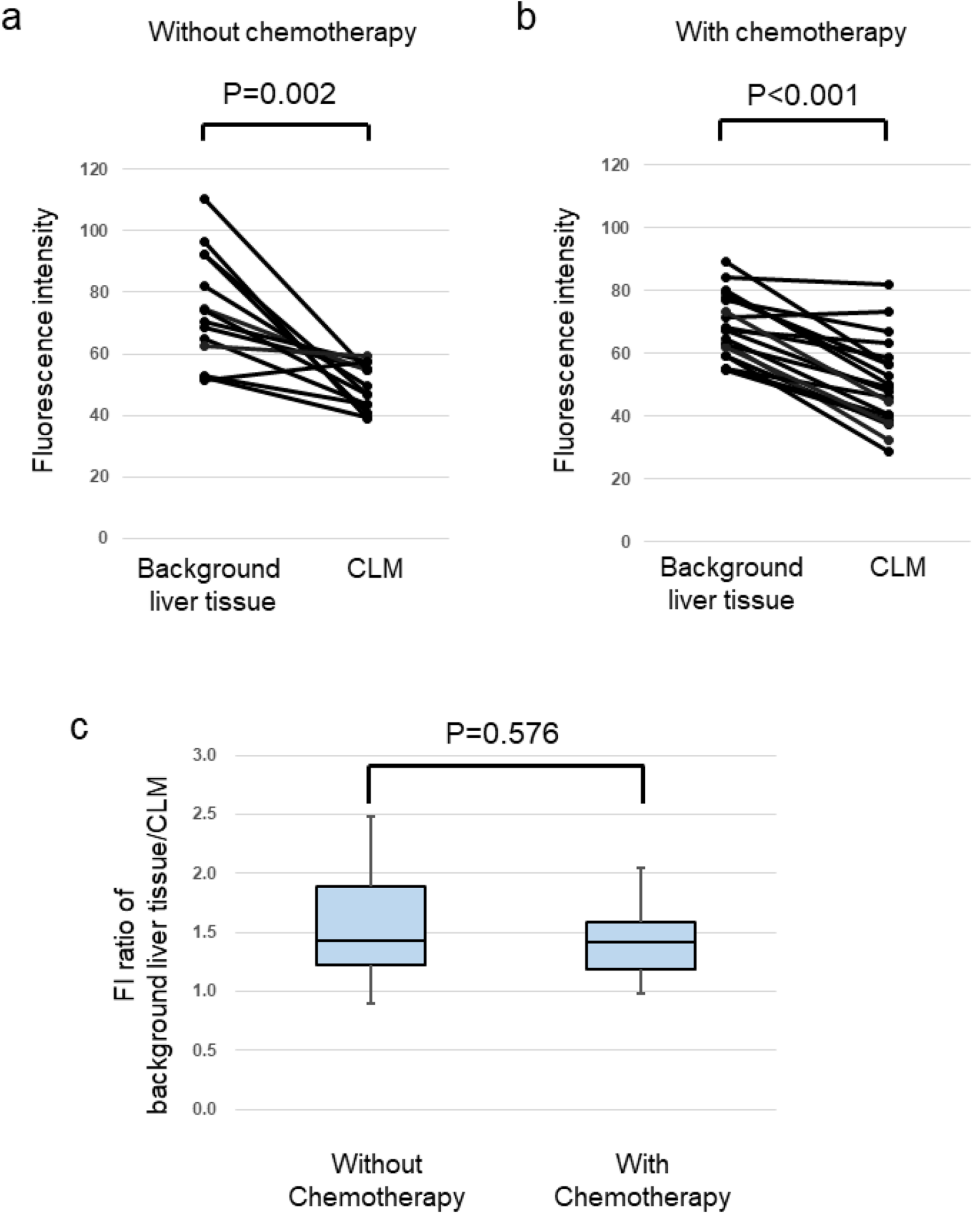
FI of cancerous and noncancerous tissue without spraying of external fluorophores. FI values were significantly lower in cancerous than in noncancerous tissue in **a** patients without prehepatectomy chemotherapy [70.4 (51.6–110) vs. 48.3 (39.0–59.4), *p* = 0.002] and **b** in patients with prehepatectomy chemotherapy [67.9 (54.6–89.2) vs. 48.6 (28.8–82.1), *p* < 0.001] **c** FI ratios (noncancerous tissue/cancerous tissue) in patients with or without prehepatectomy chemotherapy were not significantly different

Comparison of CLM and the surrounding noncancerous liver tissue found that median FI values were significantly lower in CLM than in noncancerous liver tissue in patients without prehepatectomy chemotherapy [70.4 (51.6–110) vs. 48.3 (39.0–59.4), *p* = 0.002), Fig. 3a] and in those with it [67.9 (54.6–89.2) vs. 48.6 (28.8–82.1), *p* < 0.001, Fig. 3b]. In nonenhanced pCLE images, the ratio of the background FI of noncancerous liver tissue/the FI of CLM tissue were not significantly different in patients with and without prehepatectomy chemotherapy (*p* = 0.576, Fig. 3c).

## Discussion

In nonenhanced pCLE, images of CLM and the surrounding noncancerous tissue differed in appearance and FI. Real-time pCLE of surgical specimens differentiated CLM from the surrounding noncancerous liver tissue without need of external fluorophores because of differences in tissue structure and autofluorescence. In 91.2% of the CLM tissues, nonenhanced pCLE images included tissue with an irregular structure and low autofluorescence. In 90.5% of the surgical specimens, the liver tissue surrounding the CLM had high fluorescence and a regular structure that corresponded to the regular arrangement of hepatocytes seen in HE-stained tissue. In 94.7% of the specimens, the boundary of the CLM and the surrounding liver tissue was visible. These results extend the findings of our previous report that nonenhanced pCLE distinguished malignant and healthy liver tissue in patients with primary liver tumors [13]. With the addition of an external fluorophore, pCLE visualized morphological structures that corresponded to the histopathological findings in HE-stained tissue. FS-enhanced pCLE images clearly visualized the regular arrangement of hepatocytes in healthy liver tissue, tubular structures within CLM tissue, and the boundary of CLM and noncancerous tissues. FS-enhanced pCLE images visualized changes of CLM tissue in response to prehepatectomy chemotherapy such as fibrotic structures and remaining viable cancer cells. Intravenous injection of fluorescein sodium has been used in animal models and in humans to enhance pCLE imaging of various tissues including the liver [8–12, 14, 19–23]. Fluorescein sodium is relatively safe, but intravenous injection has been associated with a 1.1–1.4% incidence of adverse effects [24, 25]. Dripping fluorescein sodium on the cut surfaces of resected tissue is intended to avoid adverse effect [15, 16]. In this study, fluorescein sodium was sprayed on the cut surfaces. One spray of fluorescein sodium was sufficient for enhanced evaluation of liver specimens. Excess fluorescein sodium hindered pCLE imaging because of blown-out highlights. The appropriate amount of fluorescein sodium needs further investigation.

Nonenhanced and FS-enhanced pCLE images of background liver tissues had a similar appearance in patients with and without prehepatectomy chemotherapy. Prehepatectomy chemotherapy causes fibrotic changes and tumor cell necrosis, resulting in the loss of tubular structures in FS-enhanced pCLE images of CLM tissue and visualization of fibrotic changes and residual cancer cells. CLM tissue following chemotherapy were differentiated in nonenhanced pCLE images because the FI ratios of noncancerous/cancerous tissue did not differ significantly in patients with or without prehepatectomy chemotherapy. pCLE was thus useful regardless of the administration of chemotherapy before surgery.

Previous studies have reported background autofluorescence in liver tissue caused by endogenous fluorophores. Nicotinamide adenine dinucleotide phosphate (NADPH), vitamin A, free fatty acids, perisinusoidal reticulin fibers, and intracellular cytokeratin are known to contribute to human liver autofluorescence [13, 26]. In this study, the FI of CLM was significantly lower than the background FI of noncancerous liver tissues, which was most likely caused by endogenous fluorophores including NADPH and vitamin A. Healthy liver tissue has previously been reported to contains more endogenous fluorophores than CLM tissue [26].

A study limitation was the lack of estimating the sensitivity and specificity of pCLE images for showing the border of cancerous tissues, which is needed to determine whether pCLE can be used to find surgical margins. Another limitation is that pCLE examination was not performed in the operative field before and during resection. The ability to visualize cancerous tissues in the operative field remains unknown.

In conclusion, pCLE permitted real-time differentiation of cancerous CLM tissue from surrounding noncancerous tissues on the basis of differences in tissue structure and FI. Fluorescein-spraying facilitated clear visualization of the morphology of CLM tissues and noncancerous liver tissues.

### Supplementary Information

Below is the link to the electronic supplementary material.

Supplementary file1 (DOC 448 KB)

## Data Availability

The data generated and analyzed during this study is available from the corresponding author on reasonable request.
