# Supplementary material for: Probe-based confocal laser endomicroscopy for real-time evaluation of colorectal liver metastasis in resected surgical specimens
Source: Hum Cell. 2023 Aug 23;36(6):2066–73. doi: 10.1007/s13577-023-00965-9 (PMC10587259; doi:10.1007/s13577-023-00965-9)

**Supplementary figure captions**

**Supplementary Fig. 1**

FS-enhanced pCLE images in (a) background liver tissue and (b) CLM tissue showing blown-out highlights


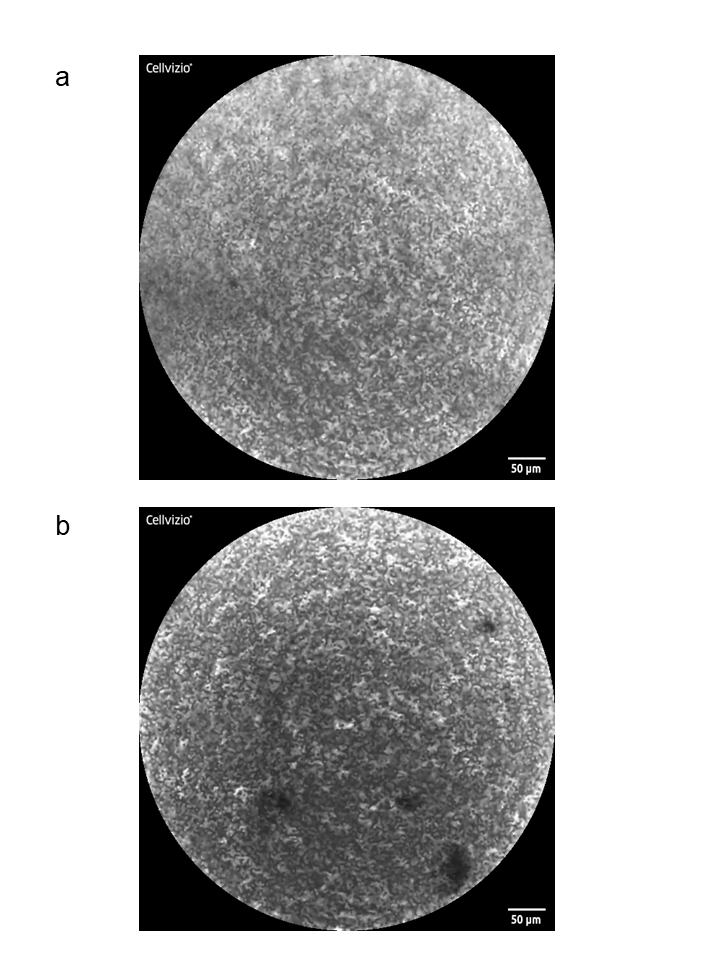

Supplement: Supplementary file 1 — Supplementary file1 (DOC 448 KB) [file 13577_2023_965_MOESM1_ESM.doc]
